# The Incidence of Steroid-Induced Hyperglycemia in Pediatric Patients With Acute Lymphocytic Leukemia

**DOI:** 10.7759/cureus.90556

**Published:** 2025-08-20

**Authors:** Abdullah Alasiri, Afnan Alamoudi, Amal Albalawi, Shahad Almatrafi, Mohammed Ameen, Khalid Alkanhal

**Affiliations:** 1 Department of Pediatric Hematology and Oncology, King Fahad Medical City, Riyadh, SAU; 2 Department of Pediatric Endocrinology, King Fahad Medical City, Riyadh, SAU; 3 Department of General Pediatrics, King Fahad Medical City, Riyadh, SAU

**Keywords:** all therapy, corticosteroid therapy, dexamethasone, pediatric acute lymphoblastic leukemia, pediatric all patients, prednisolone, steroid-induced hyperglycemia

## Abstract

Background: Steroid-induced hyperglycemia (SIH) is a frequent but often overlooked complication in pediatric patients with acute lymphoblastic leukemia (ALL). Corticosteroid use, while essential for treatment, may result in transient or persistent elevations in blood glucose. This study aimed to determine the incidence of SIH and identify potential risk factors.

Methods: A retrospective cohort study was conducted on 91 pediatric ALL patients receiving corticosteroids. Hyperglycemia was defined as blood glucose ≥200 mg/dL on at least two occasions, and borderline/transient hyperglycemia as levels between 140 and 199 mg/dL. Blood glucose was monitored throughout therapy, and demographic, clinical, and treatment variables were analyzed for associations with SIH.

Results: Among the 91 patients, 11 (12.1%) developed hyperglycemia and 12 (13.2%) had transient/borderline hyperglycemia, while 68 (74.7%) remained normoglycemic. The onset of SIH occurred between days 2 and 16 of steroid therapy, with peaks on days 4 and 10. Patients receiving dexamethasone (82.4%) had slightly higher rates of hyperglycemia compared to those on prednisolone (17.6%), though not statistically significant (p = 0.663). Similarly, no significant associations were observed with age, gender, body mass index (BMI), or risk stratification. Higher steroid doses showed a trend toward increased risk, but without statistical significance (p = 0.985).

Conclusion: SIH occurred in approximately 1 in 8 pediatric ALL patients, though no significant risk factors were identified. These findings underscore the importance of routine glucose monitoring during corticosteroid therapy, regardless of patient demographics or regimen. Tailored surveillance and early intervention protocols are recommended to mitigate potential complications and improve clinical outcomes.

## Introduction

Steroid-induced hyperglycemia (SIH) refers to elevated blood glucose levels that occur during or after corticosteroid administration, and it is a recognized complication in pediatric patients undergoing therapy for acute lymphoblastic leukemia (ALL). SIH can negatively affect both short-term patient care, by increasing infection risk and complicating chemotherapy protocols, and long-term outcomes, including heightened risks for cardiovascular, renal, and ocular complications [1-3].

The use of corticosteroids is essential in ALL treatment; however, their metabolic side effects have been well-documented. Previous research has consistently shown that corticosteroid use, especially when combined with other agents such as L-asparaginase, increases the likelihood of transient or persistent hyperglycemia [1,2]. Severe complications such as diabetic ketoacidosis have also been reported, highlighting the clinical seriousness of SIH [3]. More broadly, endocrine side effects, including hyperglycemia, are common during ALL therapy, as outlined by Howard and Pui (2002), who emphasized the multifactorial nature of treatment-related metabolic disturbances [4]. Subsequent studies, such as Gregoriou et al. (2020), further identified age and obesity as predictive risk factors and recommended insulin therapy for children developing hyperglycemia [5]. Collectively, these findings highlight that SIH is a predictable yet under-monitored treatment complication in pediatric ALL.

Recent evidence also suggests that treatment-related factors and patient characteristics influence the timing and severity of SIH. For example, Pollock et al. (2022) reported that hyperglycemia can occur shortly after administration of agents like pegaspargase, with predictors including older age, female sex, higher BMI, and family history of diabetes [6]. Similarly, other studies point to weight fluctuations during induction therapy as potential contributors to treatment-related metabolic changes [7]. These findings underscore the importance of early identification and targeted monitoring strategies.

In Saudi Arabia, the issue of SIH carries particular relevance. King Fahad Medical City, one of the largest tertiary care centers in the region, manages a high volume of pediatric ALL patients annually, estimated at over 150 new cases per year, making the need for robust monitoring and management protocols especially critical [8]. Despite this burden, there remains a lack of localized data on the incidence and risk factors of SIH in Saudi pediatric populations. This gap limits the development of evidence-based prevention and intervention strategies tailored to local healthcare contexts.

The present study addresses this gap by investigating the incidence and risk factors of SIH among pediatric ALL patients treated at King Fahad Medical City. By examining demographic, clinical, and treatment-related variables, this study aims to provide insights that can inform early detection protocols, guide preventive interventions, and ultimately improve both short- and long-term outcomes for children receiving ALL therapy.

## Materials and methods

Study design and setting

A retrospective cross-sectional study was conducted from January 2021 to December 2023 at King Fahad Medical City (KFMC), Riyadh, Saudi Arabia. KFMC is a major tertiary care hospital that provides comprehensive pediatric oncology services, making it an appropriate setting for this study.

Study subjects/participants

Eligible participants were pediatric patients younger than 14 years diagnosed with acute lymphoblastic leukemia (ALL) and treated with corticosteroids (dexamethasone or prednisolone) during the study period. Patients aged 14 years or older, those with other types of leukemia, those who did not receive corticosteroid therapy, patients with pre-existing diabetes mellitus type 1 or 2, and those with incomplete or inadequate medical records were excluded.

**Figure 1:**
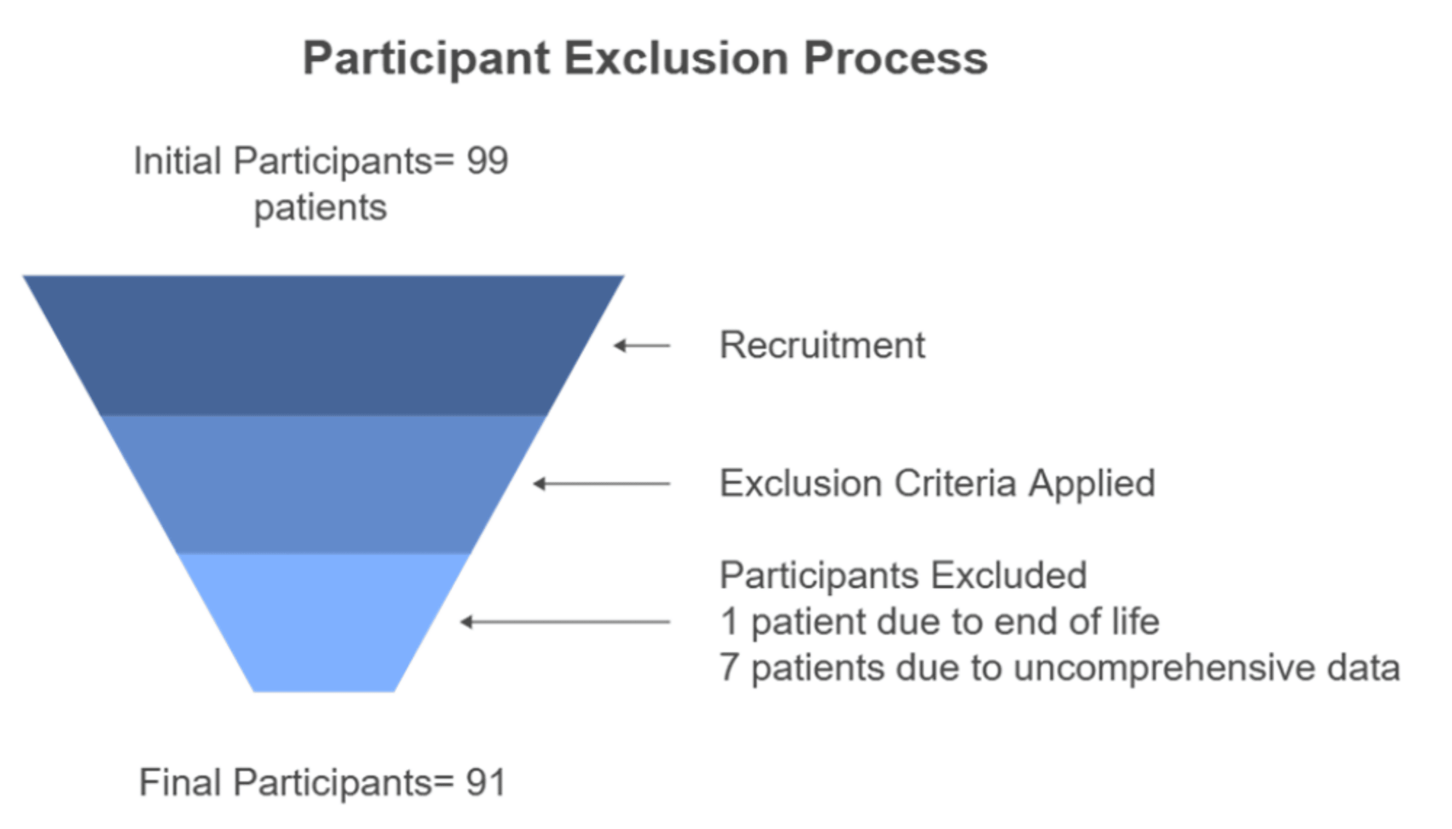
Participant exclusion process

A total of 99 potential participants were identified through the hospital’s electronic medical records (EMRs). Following exclusion, eight patients were removed (one at end-of-life and seven with insufficient data), resulting in a final study cohort of 91 patients (Figure 1).

Case definitions

Hyperglycemia was defined as two or more random blood glucose readings ≥200 mg/dL (≥11.1 mmol/L). Borderline or transient hyperglycemia was defined as glucose levels between 140 and 199 mg/dL (7.8-11.0 mmol/L). Patients not meeting these thresholds were considered normoglycemic.

Data collection

Data were extracted from KFMC’s electronic medical records (EMRs) after obtaining institutional approval. A structured data extraction form was designed in consultation with clinical experts and statisticians and pilot-tested on a subset of records. The form captured demographic data (age, gender, weight, height, and BMI), clinical details (ALL risk stratification and treatment phase), and therapeutic information (steroid type, cumulative dose, and combination with L-asparaginase). Blood glucose levels recorded during therapy were reviewed and classified according to the above thresholds.

Missing or incomplete records were excluded during the screening process, and no imputation of missing values was performed.

Ethical considerations

The study received approval from the KFMC Institutional Review Board (IRB00010471, Log No. 23-562) on October 22, 2023. Permission to access EMRs was obtained from hospital administration. Informed consent was secured from parents or guardians, and all personal identifiers were removed to preserve confidentiality.

Statistical analysis

Data were analyzed using IBM Corp. Released 2020. IBM SPSS Statistics for Windows, Version 26. Armonk, NY: IBM Corp. Descriptive statistics summarized demographic and baseline clinical data. Continuous variables were reported as mean ± standard deviation (SD) for normally distributed data or median with interquartile range (IQR) for skewed data. Categorical variables were reported as frequencies and percentages.

The incidence of SIH was calculated as a percentage of the total study cohort. Associations between SIH and categorical variables were assessed using chi-square tests. Logistic regression analysis was performed to identify independent risk factors, adjusting for age, BMI, steroid type, and cumulative dose. A p-value of <0.05 was considered statistically significant, with 95% confidence intervals (CI) reported.

## Results

Demographic and baseline characteristics

A total of 91 pediatric patients with acute lymphoblastic leukemia (ALL) who received steroid therapy were included. The mean age at diagnosis was 63.2 ± 41.9 months (approximately 5.3 ± 3.5 years). Males comprised 54 (59.3%), and females 37 (40.7%). The mean body mass index (BMI) was 15.3 ± 3.0. Based on BMI classification, 81 (89.0%) were underweight, 9 (9.9%) had normal BMI, and 1 (1.1%) was obese.

Disease type and risk stratification

High-risk B-cell ALL (HR B-ALL) and standard-risk B-ALL (SR B-ALL) were the most common categories, each observed in 30 (33.0%) patients. T-cell ALL was present in 19 (20.9%), infantile ALL in 8 (8.8%), and T-lymphoblastic lymphoma (T-LLy) in 4 (4.4%).

Genetic and molecular features

**Figure 2:**
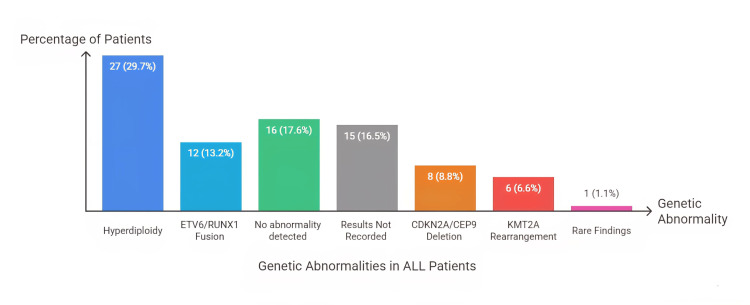
Fluorescence in situ hybridization (FISH) analysis

Fluorescence in situ hybridization (FISH) analysis revealed hyperdiploidy in 27 (29.7%) patients and ETV6/RUNX1 fusion in 12 (13.2%). A total of 16 (17.6%) had normal FISH findings, while 15 (16.5%) had results not recorded. Other abnormalities included CDKN2A/CEP9 deletion in 8 (8.8%), KMT2A rearrangement in 6 (6.6%), and rare findings such as iAMP21, IGH rearrangement, and BCR/ABL1 translocation in 1 (1.1%) patient each (Figure 2).

Steroid therapy and dosage

Dexamethasone was administered to 75 (82.4%) patients and prednisolone to 16 (17.6%). Among those on dexamethasone, 56 (61.5%) received 6 mg/m²/day and 19 (20.9%) received 10 mg/m²/day. Prednisolone dosing varied: 3 (3.3%) patients received 22.5 mg/m²/day, 8 (8.8%) received 30 mg/m²/day, 1 (1.1%) received 45 mg/m²/day, and 4 (4.4%) received 60 mg/m²/day. Most patients (64, 70.3%) received steroids for 28 days, while 19 (20.9%) received them for 14 days, and 7 (7.7%) for 7 days (Table 1).

**Table 1:** Comprehensive summary of patient characteristics, treatment, and outcomes BMI: Body mass index, FISH: Fluorescence in situ hybridization

Variable	Categories	Mean ± SD / Frequency (%)
Age at Diagnosis	Months	63.22 ± 41.87
	Years	5.3 ± 3.48
Sex	Male	54 (59.3%)
	Female	37 (40.7%)
BMI	-	15.26 ± 3.01
BMI Categories	Underweight	81 (89.0%)
	Normal	9 (9.9%)
	Obese	1 (1.1%)
Type of Disease / Risk Stratification	Infantile ALL	8 (8.8%)
	HR B-ALL	30 (33.0%)
	T-ALL	19 (20.9%)
	SR B-ALL	30 (33.0%)
	T-LLy	4 (4.4%)
FISH Results	ALL	1 (1.1%)
	BCR/ABL1	1 (1.1%)
	CDKN2A/CEP9	8 (8.8%)
	E2A/PBX1	2 (2.2%)
	ETV6/RUNX1	12 (13.2%)
	Hyperdiploidy	27 (29.7%)
	iAMP21	1 (1.1%)
	IGH	1 (1.1%)
	KMT2A	6 (6.6%)
	Normal	16 (17.6%)
	Not recorded	15 (16.5%)
Type of Steroid	Prednisolone	16 (17.6%)
	Dexamethasone	75 (82.4%)
Steroid Dose (mg/m²/day)	Dexamethasone 6	56 (61.5%)
	Dexamethasone 10	19 (20.9%)
	Prednisolone 22.5	3 (3.3%)
	Prednisolone 30	8 (8.8%)
	Prednisolone 45	1 (1.1%)
	Prednisolone 60	4 (4.4%)
Steroid Duration (Days)	7	7 (7.7%)
	14	19 (20.9%)
	28	64 (70.3%)
Hyperglycemia Status	No	68 (74.7%)
	Yes	11 (12.1%)
	Borderline/Transient	12 (13.2%)
Hyperglycemia Onset (Days)	2	3 (3.3%)
	3	4 (4.4%)
	4	5 (5.5%)
	6	3 (3.3%)
	7	2 (2.2%)
	8	2 (2.2%)
	9	1 (1.1%)
	10	4 (4.4%)
	14	1 (1.1%)
	16	1 (1.1%)

Incidence and onset of steroid-induced hyperglycemia

Hyperglycemia occurred in 11 patients (12.1%, 95% CI: 6.2-20.6%), and borderline/transient hyperglycemia in 12 patients (13.2%, 95% CI: 7.0-21.9%). The remaining 68 (74.7%) patients did not develop hyperglycemia.

**Figure 3:**
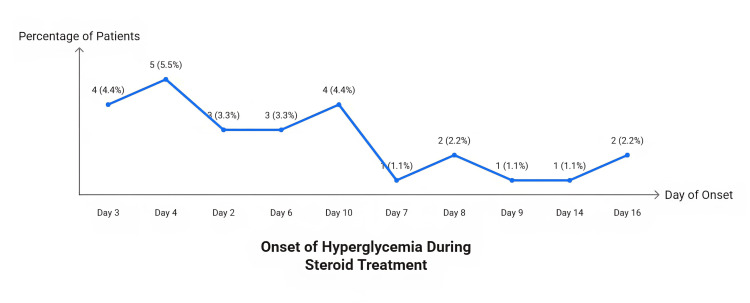
Line graph showing the onset of hyperglycemia among study participants over a 10-day period

The onset of hyperglycemia ranged from day 2 to day 16 of steroid therapy, with the highest frequency observed on days three and four (four and five patients, respectively). Other cases occurred on days two, six, and 10, while sporadic cases appeared on days seven, eight, nine, 14, and 16 (Figure 3). 

Association of hyperglycemia with sex and age

Sex-Based Differences

The relationship of hyperglycemia across sexes showed no statistically significant difference (χ² = 0.544, p = 0.762). Among male patients, seven (63.6%) developed hyperglycemia, whereas 41 (60.3%) did not. Among female patients, four (36.4%) had hyperglycemia, while 27 (39.7%) did not (Table 2). These findings suggest that gender does not play a significant role in the development of steroid-induced hyperglycemia in pediatric ALL patients.

**Table 2:** Association of various factors with hyperglycemia BMI: Body mass index

Variable	Categories	Hyperglycemia No (F%)	Hyperglycemia Yes (F%)	Chi-Square Value	p-value
Sex	Male	41 (60.3%)	7 (63.6%)	0.544	0.762
	Female	27 (39.7%)	4 (36.4%)		
Age	< 5 years	44 (64.7%)	4 (36.4%)	1.084	0.897
	5 - 10 years	24 (35.3%)	5 (45.4%)		
	> 10 years	12 (17.65%)	2 (18.2%)		
BMI	Underweight	60 (88.2%)	10 (90.9%)	0.405	0.982
	Normal	7 (10.3%)	1 (9.1%)		
	Obese	1 (1.5%)	0 (0.0%)		
Risk Stratification	Infantile ALL	7 (10.3%)	1 (9.1%)	6.227	0.622
	HR B-ALL	19 (27.9%)	5 (45.5%)		
	T-ALL	15 (22.1%)	1 (9.1%)		
	SR B-ALL	24 (35.3%)	4 (36.4%)		
	T-LLy	3 (4.4%)	0 (0.0%)		
Steroid Dose (mg/m²/day)	Dexamethasone 6.00	51 (63.75%)	5 (45.5%)	5.061	0.985
	Dexamethasone 10.00	15 (18.75%)	4 (36.4%)		
	Prednisolone 22.50	3 (3.75%)	0 (0.0%)		
	Prednisolone 30.00	7 (8.75%)	1 (9.1%)		
	Prednisolone 45.00	1 (1.25%)	0 (0.0%)		
	Prednisolone 60.00	3 (4.75%)	1 (9.1%)		
Steroid Type	Prednisolone	13 (19.1%)	2 (18.2%)	0.822	0.663
	Dexamethasone	55 (80.9%)	9 (81.8%)		
Steroid Duration	7 Days	1 (1.5%)	0 (0.0%)	10.525	0.230
	14 Days	13 (19.1%)	4 (36.4%)		
	28 Days	48 (70.6%)	7 (63.6%)		

Age-Based Differences

Age was also not significantly associated with the occurrence of hyperglycemia (χ² = 1.084, p = 0.897). A total of four (36.4%) patients under five years developed hyperglycemia, while 44 (64.7%) did not. A total of five (45.4%) patients of five to 10 years had hyperglycemia, while 24 (35.3%) remained normoglycemic. A total of two (18.2%) patients with a history of more than 10 years developed hyperglycemia, while 12 (17.65%) did not (Table 2). These results indicate that neither sex nor age group significantly influences the risk of developing steroid-induced hyperglycemia in pediatric ALL patients. However, a slightly higher percentage of cases was observed in children aged five to 10 years, demanding further investigation into potential risk factors within this age group.

Association between BMI and hyperglycemia

The relationship between body mass index and the occurrence of steroid-induced hyperglycemia was analyzed using the chi-squared test. A total of 60 (88.2%) underweight patients did not develop hyperglycemia, while 10 (90.9%) patients developed hyperglycemia. Seven (10.3%) patients of normal BMI did not develop hyperglycemia, while one (9.1%) patient developed hyperglycemia. One (1.5%) obese patient did not develop hyperglycemia. The Chi-Square value was 0.405, with a p-value of 0.982, indicating no statistically significant association between BMI and the development of steroid-induced hyperglycemia (Table 2). 

Association between risk stratification and hyperglycemia

The relationship between risk stratification and the development of steroid-induced hyperglycemia in pediatric ALL patients was analyzed using the chi-square test. The results showed no statistically significant association (χ² = 6.227, p = 0.622), indicating that the occurrence of hyperglycemia was independent of risk stratification. Among patients with infantile ALL, one (9.1%) out of eight developed hyperglycemia. In contrast, in the HR B-ALL group, five (45.5%) out of 24 were affected, indicating a relatively higher incidence in this group. Similarly, for SR B-ALL, four (36.4%) out of 28 patients developed hyperglycemia. However, in the T-ALL group, only one (9.1%) out of 16 patients experienced hyperglycemia, and no cases were reported in the T-LLy group (Table 2). Although hyperglycemia was more frequent in HR B-ALL and SR B-ALL patients, the lack of statistical significance indicates that other factors may contribute more substantially to hyperglycemia risk. Further research with larger sample sizes may help clarify any underlying associations. 

Association between steroid dose and hyperglycemia

Statistical analysis results showed no statistically significant association between steroid dose and hyperglycemia (χ² = 5.061, p = 0.985). Among patients who received dexamethasone 6 mg/m²/day, 51 (63.75%) did not develop hyperglycemia, while five (45.5%) experienced hyperglycemia. Similarly, for patients on dexamethasone 10 mg/m²/day, 15 (18.75%) remained normoglycemic, whereas four (36.4%) developed hyperglycemia. For patients who received prednisolone, none of those on 22.5 mg/m²/day (three patients) or 45 mg/m²/day (one patient) developed hyperglycemia. In contrast, one (9.1%) out of eight patients on 30 mg/m²/day and one (9.1%) out of four patients on 60 mg/m²/day developed hyperglycemia (Table 2). Despite the varying frequencies of hyperglycemia among different steroid doses, the lack of statistical significance suggests that the occurrence of hyperglycemia may not be directly dose-dependent. Other contributing factors, such as patient-specific metabolic responses, concurrent treatments, and genetic predisposition, may influence hyperglycemia risk in pediatric ALL patients undergoing steroid therapy.

Association between steroid use and hyperglycemia

Steroid Type and Hyperglycemia

Among the patients who developed steroid-induced hyperglycemia, nine (81.8%) were treated with dexamethasone, while two (18.2%) received prednisolone. Similarly, among patients who did not develop hyperglycemia, 55 (80.9%) were on dexamethasone, and 13 (19.1%) were on prednisolone. The chi-square analysis revealed no significant association between the type of steroid used and the development of hyperglycemia (χ² = 0.822, p = 0.663), suggesting that steroid type alone may not be a primary determinant of hyperglycemia risk in this cohort (Table 2).

Steroid Duration and Hyperglycemia

The duration of steroid therapy varied among patients, with most receiving steroids for 28 days. Among hyperglycemic patients, seven (63.6%) received steroids for 28 days, four (36.4%) for 14 days, and none for seven days. Comparatively, among those who did not develop hyperglycemia, 48 (70.6%) were on steroids for 28 days, 13 (19.1%) for 14 days, and one (1.5%) for seven days. The chi-square analysis showed no statistically significant association between steroid duration and hyperglycemia (χ² = 10.525, p = 0.230), though a higher proportion of hyperglycemic cases occurred in patients with prolonged steroid exposure (Table 2). These findings suggest that while both steroid type and duration contribute to the risk of hyperglycemia, neither factor had a statistically significant impact in this study. Further research into a larger sample size may be needed to clarify these associations.

## Discussion

Steroid-induced hyperglycemia (SIH) is a significant concern in pediatric patients undergoing treatment for acute lymphoblastic leukemia (ALL), as corticosteroids are a key component of therapy [8,9]. Our study aimed to evaluate the incidence of SIH in pediatric patients with ALL and assess its association with various clinical parameters, including steroid type, dosage, duration, and patient-specific characteristics. The incidence of SIH among pediatric patients with ALL and lymphoblastic lymphoma (LLy) during induction therapy in our study was 11 (12.1%). This is consistent with previous research, where the reported incidence was 9.75% when eight patients out of 82 developed hyperglycemia during the induction chemotherapy phase [10]. Another study found that 60 (21.5%) children experienced short-term hyperglycemia during their treatment for ALL among the 278 children studied [11]. A study conducted at King Abdulaziz Specialist Hospital found that 34.2% of the patients who were on steroid therapy experienced hyperglycemia [12].

The onset of hyperglycemia was most frequently observed after day three of initiating corticosteroid therapy, suggesting a cumulative steroid effect rather than an immediate response. Our study also found that the risk of hyperglycemia was higher in patients older than five years. Specifically, the incidence was greater in the five- to 10-year-old group (45.4%) compared to the >10-year-old group (18.2%). These results are consistent with prior research, which reported that 36.8% of patients aged 10 years developed hyperglycemia [10]. This trend may be attributed to age-related differences in insulin resistance, metabolic response to corticosteroids, or underlying genetic and environmental factors that influence glucose metabolism. Although the association between age and SIH was not statistically significant in our study, the lack of significance may be due to small sample size, heterogeneity of patients, or confounding effects of concurrent therapies such as L-asparaginase, which is also known to affect glucose metabolism.

Among the different risk stratification groups, patients with High-Risk B-ALL (HR-ALL) had the highest incidence of SIH 5 (45.5%), followed by Standard-Risk B-ALL (SR-ALL) 4 (36.4%). This may be due to differences in treatment intensity, as HR-ALL patients often receive higher steroid doses or prolonged corticosteroid therapy, leading to greater metabolic disturbances. Additionally, factors such as underlying disease biology, coexisting comorbidities, and higher systemic inflammation in HR-ALL patients could contribute to increased susceptibility to hyperglycemia. However, these trends were not statistically significant, which again may reflect limited statistical power in this sample.

Steroid type also appeared to influence the risk of hyperglycemia. Patients receiving dexamethasone exhibited a higher incidence of hyperglycemia, 9 (81.8%), compared to those receiving prednisolone, 2 (18.2%). These findings align with previous studies that reported dexamethasone as being more diabetogenic due to its longer half-life and higher glucocorticoid receptor affinity, which leads to prolonged suppression of insulin sensitivity. Prior studies have shown higher rates of insulin requirement among patients treated with dexamethasone compared to prednisolone [12]. Nevertheless, in our study, this difference was not statistically significant (p = 0.663), which may again be due to the relatively small sample size and variations in steroid regimen.

Moreover, there was no significant difference in the risk of hyperglycemia between male and female patients, indicating that sex may not be a substantial determinant of steroid-induced metabolic effects. This finding is similar to prior studies, in which gender did not significantly affect the incidence of hyperglycemia, though some reports noted higher percentages in females [10]. Similarly, in our study, BMI categories (underweight, normal, and obese) did not show a significant association with hyperglycemia. While obesity is a well-documented risk factor for steroid-induced diabetes in adults, the high proportion of underweight patients in our study (89.0%) may have influenced this outcome. These findings suggest that factors beyond BMI, such as individual metabolic responses, concurrent agents like L-asparaginase, and genetic predispositions, may play a more significant role in SIH development in pediatric ALL/LLy patients.

Limitations and future directions

This study has several limitations that should be considered when interpreting the findings. First, the relatively small sample size may have limited the statistical power to detect significant associations, which could explain why some observed trends (e.g., higher risk in certain age groups or with dexamethasone use) did not reach significance. Larger, multicenter studies are recommended to confirm these findings and provide more generalizable results.

Second, the retrospective design and single-center setting may restrict the external validity of the study. The reliance on existing medical records could also introduce information bias, particularly in cases where data were incomplete or missing.

Third, while we examined major clinical and treatment-related factors, we did not assess potential confounding variables such as baseline insulin resistance, family history of diabetes, or other metabolic risk factors that may influence susceptibility to steroid-induced hyperglycemia.

Finally, the relatively short study duration (three years) and lack of long-term follow-up data limited our ability to evaluate whether hyperglycemia persisted beyond steroid therapy or contributed to later complications.

## Conclusions

In this study, the incidence of steroid-induced hyperglycemia (SIH) among pediatric ALL patients was 12.1%, with the highest frequency of onset occurring between days three and four of corticosteroid therapy. Although no statistically significant associations were found with age, sex, BMI, or steroid type, trends suggested a higher risk in patients aged 5-10 years and in those treated with dexamethasone. Based on these findings, we recommend that routine glucose monitoring should begin as early as day two of corticosteroid therapy and continue throughout the induction phase, with particular attention to patients receiving dexamethasone or intensive treatment regimens. Incorporating structured glucose checks into treatment protocols could help detect SIH earlier and allow timely intervention. Finally, the absence of standardized monitoring protocols in Saudi Arabia and the broader MENA region highlights the need for locally adapted guidelines to improve early detection and management of SIH in pediatric ALL patients.
